# Glutamine Metabolism Scoring Predicts Prognosis and Therapeutic Resistance in Hepatocellular Carcinoma

**DOI:** 10.3389/pore.2021.1610075

**Published:** 2021-12-14

**Authors:** Leqian Ying, Meilian Cheng, Yi Lu, Qin Tao, Xiaofeng Chen, Bo Shen, Fen Xiong, Zhangmin Hu, Deqiang Wang, Xiaoqin Li

**Affiliations:** ^1^ Department of Medical Oncology, The Affiliated Hospital of Jiangsu University, Zhenjiang, China; ^2^ Department of Medical Oncology, Jiangsu Province Hospital, Nanjing, China; ^3^ Department of Medical Oncology, The Affiliated Cancer Hospital of Nanjing Medical University, Nanjing, China

**Keywords:** immunotherapy, prognosis, glutamine metabolism, hepatocellular carcinoma, immune checkpoint inhibitors, therapeutic resistance

## Abstract

Glutamine metabolism (GM) plays a critical role in hepatocellular carcinoma (HCC); however, a comprehensive methodology to quantify GM activity is still lacking. We developed a transcriptome-based GMScore to evaluate GM activity and investigated the association of GMScore with prognosis and therapeutic resistance. Two independent HCC cohorts with transcriptome data were selected from The Cancer Genome Atlas (TCGA, *n* = 365) and the International Cancer Genome Consortium (ICGC, *n* = 231). The expression of 41 GM-associated genes were used to construct and validate GMScore. Several genomic or transcriptomic biomarkers were also estimated. Tumor response to immune checkpoint inhibitors (ICIs) was predicted using the tumor immune dysfunction and exclusion algorithm. GMScore was closely correlated with patient characteristics, including stage, histology grade, alpha-fetoprotein level, and vascular invasion. High GMScore was an independent risk factor for overall survival (OS) in both cohorts (HR = 4.2 and 3.91, both *p* < 0.001), superior to clinical indices and other biomarkers. High GMScore presented transcriptome features to indicate cell growth advantages and genetic stability, which was associated with poor OS of patients who received transcatheter arterial chemoembolization (TACE). High GMScore was also related to high expression of immune checkpoint genes, increased infiltration of regulatory T cells, and decreased infiltration of M1 macrophages. More importantly, high GMScore indicated poor predicted responses to ICIs, which could be verified in an ICI-treated melanoma cohort. In conclusion, GMScore is a strong prognostic index that may be integrated into existing clinical algorithms. A high GMScore may indicate resistance to TACE and ICIs based on its transcriptome and immune features. Validations using other HCC cohorts, especially ICI-treated HCC cohorts, are necessary.

## Introduction

Primary liver cancer is the seventh most prevalent cancer and the second leading cause of cancer-related death in the world [1]. Hepatocellular carcinoma (HCC) is the most common form of liver cancer, accounting for approximately 90% of cases. Currently, hepatitis B and hepatitis C virus infections remain the most important global risk factors for HCC. Meanwhile, metabolic risk factors, such as obesity, metabolic syndrome, diabetes mellitus, and non-alcoholic fatty liver disease are increasingly prevalent and have become important causes of HCC [2]. However, regardless of pathogenic factors, abnormal metabolism plays a critical role in hepatocellular carcinogenesis and HCC development because the liver is a metabolic organ [3].

Aberrant active glutamine metabolism (GM) is a key player in HCC. The tumorigenicity of HCC stem cells was inhibited by knocking out glutaminase 1 (GLS1), with a high expression of GLS1 predicting poor prognosis [4]. Moreover, the expression of glutamine transporter ASCT2 was significantly upregulated in HCC and was an independent prognostic risk factor [5]. In contrast, high expression of oxoglutarate dehydrogenase-like, which limited GM, was associated with favorable prognosis of HCC patients and sensitized HCC cells to sorafenib [6]. These findings indicate that the expression levels of GM-related genes reflect the molecular heterogeneity of HCC, which determines distinct clinical outcomes of HCC patients. However, there is still a lack of models to quantify GM activity based on relevant gene expressions whose acquisition has been largely promoted by next-generation sequencing (NGS).

In this study, we established a transcriptome-based methodology named GMScore to quantify GM activity in HCC. We showed that GMScore was a predictor not only for prognosis but also for treatment outcomes in two independent HCC cohorts.

## Materials and Methods

### Patients and Data Collection

We screened patients from The Cancer Genome Atlas (TCGA) as the discovery set and patients from the International Cancer Genome Consortium (ICGC) as the validation set. The following enrollment criteria were used: 1) available sequencing data for GMScore calculation, 2) pathological diagnosis of HCC, and 3) no prior history of radiation therapy, chemotherapy, target therapy, immunotherapy, or other anticancer medications (including neoadjuvant therapy). Sequencing data and corresponding clinical information, recorded up to August 1, 2021, were downloaded from TCGA (https://portal.gdc.cancer.gov/repository) and ICGC (https://dcc.icgc.org/projects/LIRI-JP) data portals. An additional melanoma cohort (GSE78220) treated with anti-PD-1 was used to verify the association of GMScore with immunotherapy outcomes, and data were downloaded from Gene Expression Omnibus (GEO) [7]. Genetic expression data presented as the fragments per kilobase per million (FPKM) values were transformed into transcripts per kilobase million (TPM) values to improve comparability between samples [8]. The American Joint Committee on Cancer criteria was used for clinical and clinicopathological classification and staging.

### GMScore Construction

The 41 GM-related genes (Supplementary Table S1) were extracted from the Gene Ontology (GO) initiative and a published study [9]. Of them, differentially expressed genes between cancerous and the adjacent normal tissues were identified by the *limma* R package in HCC patients of TCGA, with a false discovery rate of <0.05. Subsequently, genes associated with overall survival (OS) were selected using univariate Cox regression models. The optimal cut-off values to define high and low expression subgroups were determined based on the association of genetic expression with OS using the *Survminer* R package. Subsequently, gene expression level was evaluated as 0 or 1; a value of 0 was assigned when the gene expression was less than the corresponding cut-off value, and a value of 1 otherwise. Furthermore, the least absolute shrinkage and selection operator (LASSO) Cox regression model was used to screen the most useful prognostic genes. A GMScore model was then constructed based on the fraction of selected genes using Cox regression coefficients. The formula was established as follows: GMScore = sum (each gene’s expression × corresponding coefficient).

### Immune, Stromal, and ESTIMATE Scoring

The ESTIMATE algorithm was used to estimate the relative fraction of stromal and immune cells in the tumor microenvironment (TME) and was exhibited in the form of the StromalScore, ImmuneScore, and ESTIMATEScore. Of these, ESTIMATEScore correlated with DNA copy number-based tumor purity [10].

### Tumor Mutation Burden Calculation

The total counts of somatic nonsynonymous variations, including missense, nonsense, splice-site, and frameshift mutations, in coding regions were defined as TMB [11].

### Gene Set Enrichment Analysis

Differentially expressed genes between the low and high GMScore subgroups were determined, with the criteria of adjusted *p*-value <0.05 and log2 (fold change) >1, and GSEA based on GO and Kyoto Encyclopedia of Genes and Genomes (KEGG) was performed using NetworkAnalyst 3.0 [12].

### Immune Infiltration Estimation

The CIBERSORT algorithm and the LM22 gene signature were utilized to quantify the abundance of infiltrating immune cells in the TME, based on transcriptome data [13].

### Tumor Immune Dysfunction and Exclusion Scoring

TIDE, a computational method based on transcriptome to model the induction of T cell dysfunction and the prevention of T cell infiltration in tumor immune evasion, was used to calculate T cell dysfunction and exclusion scores, which were further merged as the TIDE score to predict tumor response to immune checkpoint inhibitors (ICIs) [14].

### Statistical Analysis

R software (version 4.0.3, http://www.r-project.org) or IBM SPSS Statistics ver.20 (IBM Corp., Armonk, NY, United States) were used to analyze the related data and plot graphs. The Student’s *t*-test, chi-square test, Fisher’s exact probability test, or Mann-Whitney U test were used to compare the differences between groups. The Kaplan-Meier method with the log-rank test was used to compare OS between different parts. Hazard ratios (HRs) and their 95% confidence intervals (CIs) for prognostic factors were calculated using univariate and multivariate Cox proportional hazard models. Patients were separated into high and low GMScore groups based on the optimal value associated with OS calculated using the *Survminer* R package. Receiver operating characteristic curve (ROC), time-dependent ROC curves, and the areas under the ROC curves (AUC) depicted or calculated by the *timeROC* R package, were used to evaluate the predictive power of the models. Statistical significance was set at *p* < 0.05, and all *p*-values were two-tailed.

## Results

### Patient Characteristics

A total of 365 patients from TCGA and 231 patients from ICGC were included (Supplementary Table S2). Patients in ICGC were older and had more stage III/IV diseases than those in TCGA (*p* < 0.05). Histology grade, alpha-fetoprotein (AFP), and vascular invasion data were only available in TCGA.

### Derivation of the GMScore and its Association With Clinical Features

**FIGURE 1:**
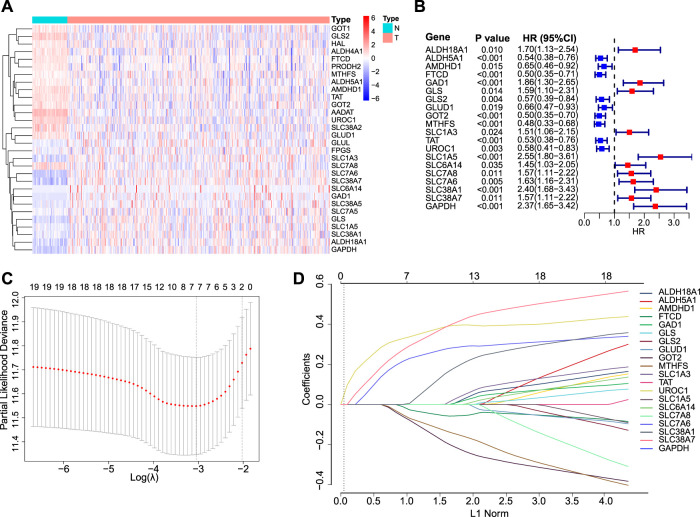
GMScore construction. **(A)**: Heatmap for 30 differentially expressed GM-associated genes between tumor (T) and normal tissues (N) in the TCGA HCC cohort. **(B)**: Forest plot showing hazard ratios of 20 genes in A which were significantly associated with overall survival of HCC patients from TCGA. **(C)**: Tenfold cross-validation for tuning parameter selection in the LASSO model. The minimum standard was accepted to obtain the value of the super parameter *λ* in the LASSO-Cox model. The *λ* value was confirmed as 0.03934 where the optimal *λ* resulted in 7 non-zero coefficients. **(D)**: LASSO coefficient profiles of the fractions of 20 genes in **(B)**. GM, glutamine metabolism; TCGA, The Cancer Genome Atlas; HCC, hepatocellular carcinoma; LASSO, least absolute shrinkage and selection operator.

A total of 30 GM-related genes were differentially expressed between HCC and normal tissues (Figure 1A). Of these, 20 genes were significantly associated with OS (Figure 1B). After LASSO Cox regression analysis (Figures 1C,D), seven genes were selected to construct the GMScore of OS, as follows: GMScore = 0.374 ∗ expression level of SLC1A5 + 0.359 ∗ expression level of GAPDH +0.264 ∗ expression level of SLC38A1 + 0.112 ∗ expression level of SLC38A7 − 0.049 ∗ expression level of FTCD-0.113 ∗ expression level of MTHFS-0.157 ∗ expression level of GOT2.

Patients with high GMScores in both TCGA and ICGC included a significantly higher proportion of patients with stage III/IV disease (*p* < 0.001 and *p* = 0.009, respectively) than those with low GMScores. Histology grade 3/4 (*p* < 0.001), AFP larger than 200 ng/ml (*p* < 0.001), and vascular invasion (*p* = 0.001) were also more frequent in patients with high than in those with low GMScores (Table 1).

**TABLE 1:** Patient characteristics according to GMScore level.

Characteristic	TCGA			ICGC (%)		
	High (%)	Low (%)	*p* Value	High (%)	Low (%)	*p* Value
Age						
≤65 years	91 (64.1)	136 (61.0)	0.552	46 (38.7)	43 (38.4)	0.967
>65 years	51 (35.9)	87 (39.0)		73 (61.3)	69 (61.6)	
Gender						
Female	51 (35.9)	68 (30.5)	0.281	33 (27.7)	28 (25.0)	0.638
Male	91 (64.1)	155 (69.5)		86 (72.3)	84 (75.0)	
Stage						
I + II	87 (61.3)	167 (74.9)	<0.001	63 (52.9)	78 (69.6)	0.009
III + IV	49 (34.5)	38 (17.0)		56 (47.1)	34 (30.4)	
Unknown	6 (4.2)	18 (8.1)		0 (0)	0 (0)	
Histology grade						
G1 + G2	72 (50.7)	158 (70.9)	<0.001	NA	NA	
G3 + G4	68 (47.9)	62 (27.8)		NA	NA	
Unknown	2 (1.4)	3 (1.3)		NA	NA	
Alpha-fetoprotein						
≤200 ng/ml	58 (40.8)	143 (64.1)	<0.001	NA	NA	
>200 ng/ml	38 (26.8)	37 (16.6)		NA	NA	
Unknown	46 (32.4)	43 (19.3)		NA	NA	
Vascular invasion						
No	64 (45.1)	141 (63.2)	0.001	NA	NA	
Yes	47 (33.1)	59 (26.5)		NA	NA	
Unknown	31 (21.8)	23 (10.3)		NA	NA	

TCGA, The Cancer Genome Atlas; ICGC, International Cancer Genome Consortium; NA, not available.

### GMScore is an Independent Prognostic Factor

**FIGURE 2:**
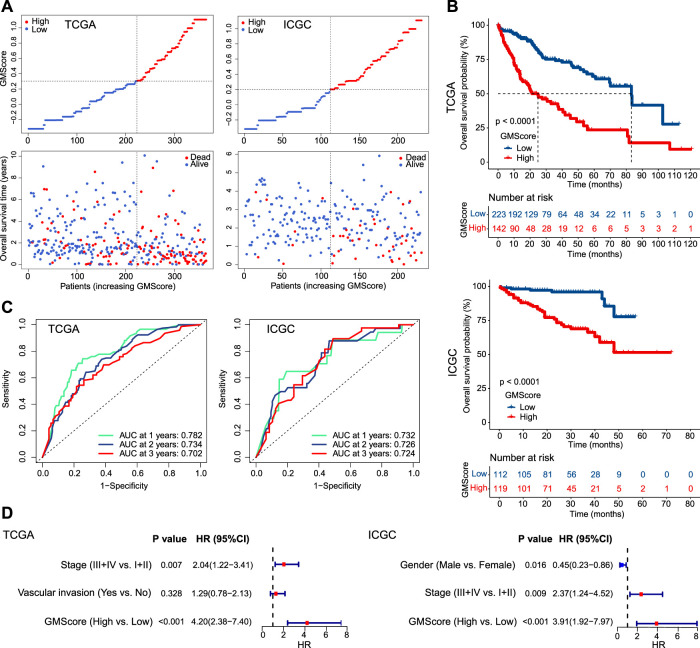
GMScore and prognosis. **(A)**: Models of prognostic prediction dividing patients into high and low risk groups, and the cut-off value for high and low GMScore was determined. **(B)**: High GMScore significantly correlated with poor overall survival (OS). **(C)**: Time-dependent ROC curves evaluated the predictive ability of GMscore for 1-year, 2-years and 3-years OS. **(D)**: High GMScore was an independent risk factor for OS in multivariate Cox regression models (variables were selected by univariate Cox regression models). GM, glutamine metabolism; TCGA, The Cancer Genome Atlas; ICGC, International Cancer Genome Consortium; ROC, receiver operating characteristics; AUC, area under the curve; HR, hazard ratios; CI, confidence interval.

Patients were divided into high and low GMScore subgroups to establish a prognosis-predictive model in TCGA, which was validated using ICGC (Figure 2A). Kaplan-Meier survival analysis confirmed the survival discrepancy between high and low GMScore groups (Figure 2B), and the results of the ROC curve analysis verified the predictive value of the established risk model in both TCGA and ICGC (Figure 2C). After univariate selection for prognostic significance of variables (Supplementary Figure S1), multivariate analysis in both TCGA and ICGC showed that high GMScore was an independent predictor of poor OS (HR = 4.2, 95% CI 2.38–7.4, *p* < 0.001, and HR = 3.91, 95% CI 1.92–7.97, *p* < 0.001, respectively), even superior to staging in terms of HR (Figure 2D).

### GMScore is Superior Than Other Biomarkers for Prognostic Prediction

**FIGURE 3:**
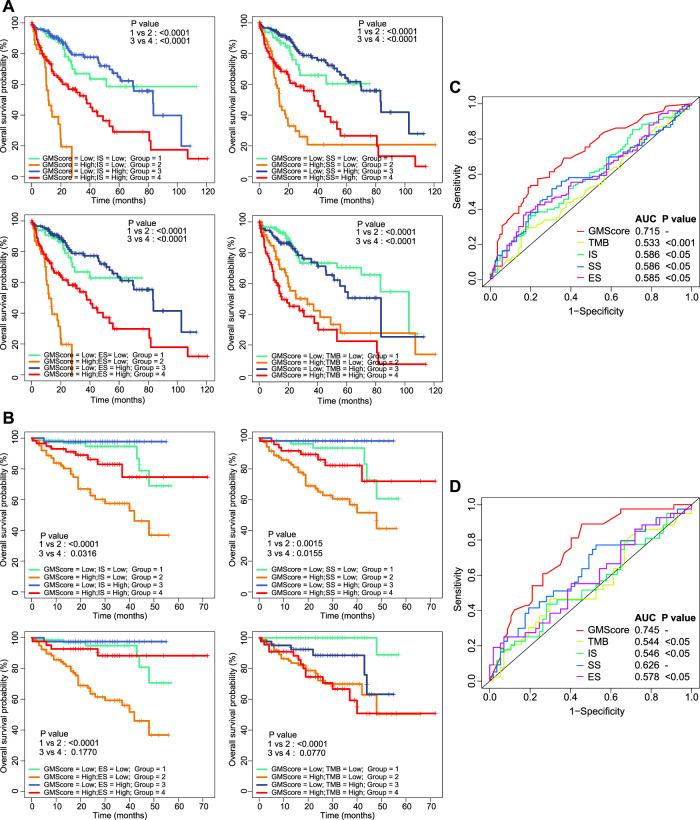
Other biomarkers and prognosis, stratified by GMScore. **(A)** and **(B)**: Overall survival (OS) in both high and low subgroups of ImmuneScore, StromalScore, EstimateScore, and TMB could be further stratified by the GMScore level in HCC patients from TCGA **(A)** and ICGC **(B)**. **(C)** and **(D)**: Time-dependent ROC curves evaluated the predictive ability of GMscore and other biomarkers for 3-years OS in both TCGA **(C)** and ICGC **(D)**. TMB, tumor mutation burden; TCGA, The Cancer Genome Atlas; ICGC, International Cancer Genome Consortium; ROC, receiver operating characteristics; AUC, area under the curve; SS, StromalScore; IS, ImmuneScore; ES, ESTIMATEScore.

ImmuneScore, StromalScore, ESTIMATEScore, and TMB have been reported as prognostic predictors in many cancers [10, 11, 15]. We showed that they were also associated with or tended to be associated with OS of HCC (Supplementary Figure S2). However, prognostic stratification according to these factors could be further optimized using GMScore. The OS of both patients in the high and low subgroups of these biomarker values could be further stratified by the GMScore level (Figures 3A,B). Moreover, the ROC curve analysis revealed that GMScore was better than other biomarkers in terms of the prediction of 3-years OS (only two patients were followed up for over 5 years in ICGC) (Figures 3C,D).

### Transcriptome Features of High GMScore Indicate Therapeutic Resistance

**FIGURE 4:**
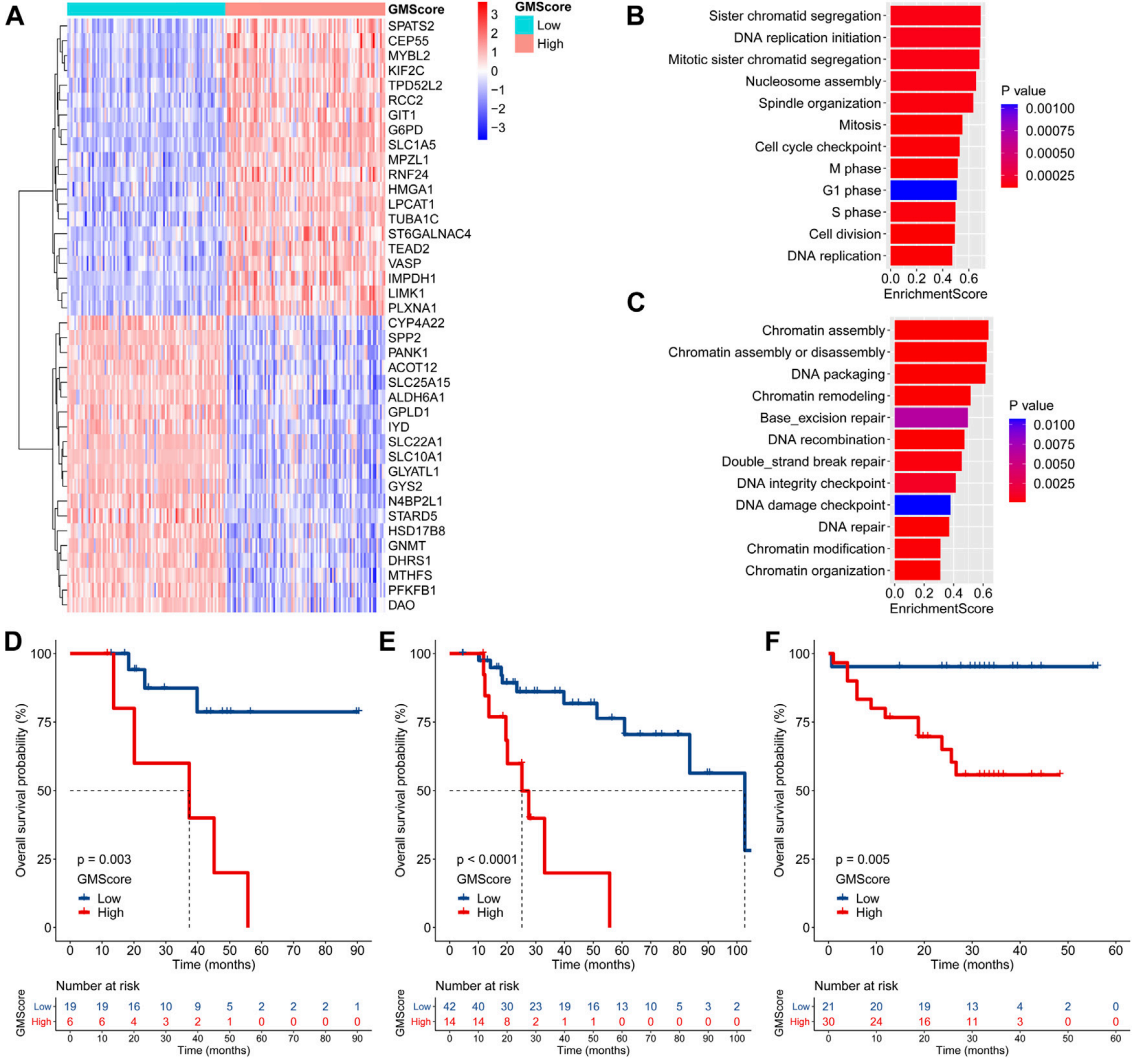
GMScore and therapeutic resistance. **(A)**: Heatmap for top 40 differentially expressed genes between high and low GMScore subgroups. **(B)**: Selected GO BP terms for cell proliferation and cell cycle **(B)**, and DNA repair and chromatin regulation **(C)** in GSEA. **(D)** and **(E)**: Overall survival (OS) of patients received adjuvant **(D)** or salvage **(E)** transcatheter arterial chemoembolization (TACE), stratified by GMScore level in the TCGA HCC cohort. **(F)**: OS of patients received TACE stratified by GMScore level in the ICGC HCC cohort (timing was unknown). GO, Gene Ontology; BP, biological process; GSEA, gene set enrichment analysis; TCGA, The Cancer Genome Atlas; ICGC, International Cancer Genome Consortium; HCC, hepatocellular carcinoma.

Differentially expressed genes between the high and low GMScore subgroups were determined (Figure 4A and Supplementary Table S3). GSEA based on the GO biological process (BP) and KEGG pathways was performed (Supplementary Table S4). We revealed that genes associated with proliferation and cell cycle were significantly enriched in the high GMScore subgroup, indicating that tumors with high GMScores might have cell growth advantages (Figure 4B). Meanwhile, high GMScore also enriched genes associated with DNA repair and chromatin regulation, suggesting that tumors with high GMScores may be characterized by genomic stability (Figure 4C).

It is known that genomically stable or elevated DNA repair correlates with therapeutic resistance; thus, we examined the impact of high GMScore on outcomes of transcatheter arterial chemoembolization (TACE). In the TCGA HCC cohort, high GMScore significantly decreased OS in patients who received adjuvant TACE after surgery (*p* = 0.003; Figure 4D) or salvage TACE after recurrence (*p* < 0.001; Figure 4E) than those with low GMScore. A similar result was found in the ICGC HCC cohort (Figure 4F), although the timing of TACE was unknown.

### GMScore is Associated With Immune Profiles and May Predict Response to ICIs

**FIGURE 5:**
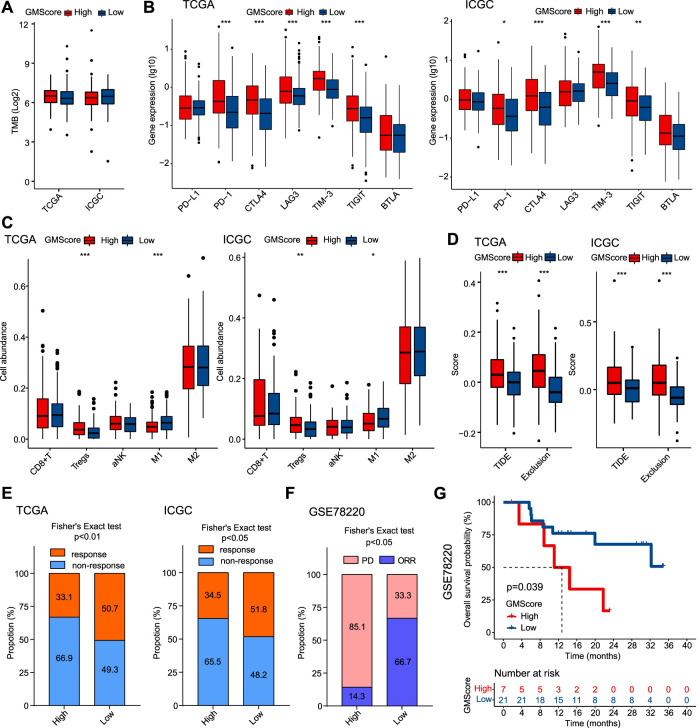
GMScore and immune profiles and immunotherapy efficacy. **(A)**: Relationship between GMScore and TMB; **(B)**: GMScore and the expressions of immune checkpoint genes. **(C)**: GMScore and the infiltration of selected immune cells in tumors determined by the CIBERSORT algorithm. **(D)**: GMScore and exclusion score and TIDE score. **(E)**: GMScore and the predicted response to immune checkpoint inhibitors by the TIDE algorithm. **(F)**: GMScore and the response to anti-PD-1 therapy in a melanoma cohort (GSE78220). **(G)**: GMScore and overall survival of patients in the GSE78220 cohort. TMB, tumor mutation burden; TCGA, The Cancer Genome Atlas; ICGC, International Cancer Genome Consortium; TIDE, Tumor Immune Dysfunction and Exclusion; PD, progression disease; ORR, objective response rate.

TMB has been identified as a predictor of ICI efficacy in multiple cancers. In this study, we found that GMScore level did not affect TMB (Figure 5A). However, high GMScore subgroup had significantly higher gene expressions than low GMScore subgroup in terms of several immune checkpoints, including PD-1, CTLA-4, TIM-3, and TIGIT in both TCGA and ICGC (Figure 5B). For immune cell infiltration in tumors, high GMScore subgroup had a significantly higher abundance of regulatory T cells (Tregs) but a significantly lower abundance of M1 macrophages than low GMScore subgroup (Figure 5C). These findings indicate that GMScore may partly reflect TME.

Because no HCC cohorts treated by ICIs with transcriptome data were available, we used the TIDE algorithm to calculate T cell dysfunction and exclusion scores, and predict ICI response by the merged TIDE score. We observed that high GMScore subgroup had significantly higher exclusion score and TIDE score compared to the low GMScore subgroup (Figure 5D). Consequently, for the predicted response rate, high GMScore HCC was significantly inferior to low GMScore HCC in both TCGA and ICGC (Figure 5E). Furthermore, we used an ICI-treated melanoma cohort to verify the impact of GMScore on immunotherapy outcomes and found that the high GMScore subgroup had a significantly lower objective response rate than the low GMscore subgroup (14.3 vs. 66.7%, *p* = 0.016; Figure 5F). More importantly, high GMScore significantly reduced OS compared to low GMScore (*p* = 0.039; Figure 5G).

## Discussion

This is the first study to evaluate GM activity by quantifying GM-associated gene expression as the GMScore in HCC. We revealed that GMScore was an independent OS predictor superior to clinical indices and other biomarkers. HCC with high GCS scores may be genomically stable and resistant to TACE. In addition, GMScore was associated with immune profiles and may be helpful in the prediction of response to ICIs. Together, the methodology of GM evaluation, such as ours, may improve the understanding and clinical outcomes of HCC.

In the recent decade, improvements in therapeutic approaches have been achieved in HCC. In contrast, there has not been any significant clinical improvement in HCC biomarkers in predicting prognosis and treatment response. Some existing clinical algorithms that have been proposed for HCC do not include biomarkers, such as the Barcelona Clinic Liver Cancer (BCLC) system, the Japan Integrated Scoring (JIS) system, and the Hong Kong Liver Cancer (HKLC) system. On the other hand, other existing clinical algorithms, such as the albumin-bilirubin (ALBI) grade and the BALAD score, include only traditional biochemical parameters such as AFP, serum bilirubin, and albumin [16]. Novel biomarkers, especially those developed by NGS, such as genomic and transcriptome features, are still far from clinical application, although increasing evidence indicates their critical impacts on clinical outcomes of HCC [17, 18]. In this study, GMScore had a stronger prognostic effect on HCC than staging, which was independent of clinical indices. GMScore also has potential influences on treatment outcomes. Further studies are needed to test whether GMScore can be integrated with existing clinical algorithms to further improve clinical practice.

GMScore allowed us to explore GM-associated transcriptome features. It is reasonable to observe an association between high GMScore and cell growth advantages indicated by GSEA, because glutamine is an essential nutrient for cancer cell proliferation. Interestingly, high GMScore may improve DNA repair and genomic stability, which may induce TACE-resistance in both the TCGA and ICGC HCC cohorts. Many pieces of evidence support our findings. Glutamine deficiency induces DNA alkylation damage and DNA damage accumulation to trigger genomic instability and hyposensitize cancer cells to alkylating agents [19]. Glutamine synthetase (GS), an enzyme catalyzing glutamate and ammonia to glutamine, improves DNA repair and causes radiation resistance [20]. Moreover, high GS expression decreased OS and increased early phase recurrence in 554 HCC patients from two independent cohorts who underwent adjuvant TACE [21].

The deleterious role of GM in anticancer immunity is intriguing. Glutamine blockade suppressed cancer cells but induced a long-lived, highly activated phenotype for effector T cells [22]. Targeting GM rendered ICI-resistant tumors susceptible to immunotherapy by modulating myeloid-derived suppressor cells (MDSCs) in a breast cancer model [23]. In combination with anti-PD-L1, glutamine depletion in mice strongly promoted antitumor efficacy of T cells by increasing Fas/CD95 levels [24]. Our study revealed that high GMScore may promote the genetic expression of immune checkpoints and the infiltration of immunosuppressive Tregs, but impede the infiltration of immunoactivated macrophages M1. Similarly, a previous study on kidney cancer also identified a glutamine signature (GlnS) to show that high-GlnS tumors had higher levels of Tregs and impaired T-cell cytotoxic function [9]. However, the impact of GM on immunotherapy efficacy has not been investigated in cancer patients. In this study, we predicted the tumor response to ICIs in patients with HCC using the well-established TIDE algorithm. We showed that GMScore may be associated with such a response, which could be validated in an ICI-treated melanoma cohort. These findings further support the development of GM inhibitors in clinical trials and GM evaluation in the efficacy prediction of immunotherapy.

This study has several limitations. First, more HCC patients are necessary to validate the accuracy of prognosis prediction based on GMScore. Second, the findings of this study have not been verified experimentally. Moreover, predictions rather than true tumor responses to ICIs in HCC patients were used, although the impact of GMScore on immunotherapy was confirmed in melanoma.

In conclusion, we showed that GMScore, a multigene model related to GM, could predict both prognosis and therapeutic resistance in HCC. In particular, GMScore may impact immunotherapy response, which is highly interesting considering the recent arrival of the immunotherapy era in HCC. These results will help in understanding of the cancer biology, stratifying the prognosis, developing precise medical strategies, and improving the survival of HCC patients. Our study stressed that GM evaluation and GM blockade could be an interesting area for further investigation.

## Data Availability

Publicly available datasets were analyzed in this study. This data can be found here: HCC data in our study is available in TCGA (https://portal.gdc.cancer.gov/repository) and ICGC (https://dcc.icgc.org/projects/LIRI-JP) data portals. The data of ICI-treated melanoma cohort is available in Gene Expression Omnibus under accession number GSE78220 (https://www.ncbi.nlm.nih.gov/geo/query/acc.cgi?acc=GSE78220).

## Supplementary Material

Supplementary Figure S1 Univariate Cox regression analyses of variables. **(A)**: The Cancer Genome Atlas (TCGA) cohort. **(B)**: The International Cancer Genome Consortium (ICGC) cohort. HR, hazard ratios; CI, confidence interval.

Supplementary Figure S2 Other biomarkers and prognosis. **(A)**: The Cancer Genome Atlas (TCGA) cohort. **(B)**: The International Cancer Genome Consortium (ICGC) cohort. TMB, tumor mutation burden.

## Abbreviations

ALBI, albumin-bilirubin; AFP, alpha-fetoprotein; AUC, area under the ROC curves; BCLC, Barcelona Clinic Liver Cancer; CIs, confidence intervals; GO, Gene Ontology; GSEA, gene set enrichment analysis; GLSI, glutaminase 1; NGS, next-generation sequencing; GM, glutamine metabolism; GlnS, glutamine signature; GS, glutamine synthetase; HRs, hazard ratios; HCC, hepatocellular carcinoma; HKLC, Hong Kong Liver Cancer; ICIs, immune checkpoint inhibitors; ICGC, International Cancer Genome Consortium; JIS, Japan Integrated Scoring; KEGG, Kyoto Encyclopedia of Genes and Genomes; LASSO, least absolute shrinkage and selection operator; MDSCs, myeloid-derived suppressor cells; OS, overall survival; ROC, receiver operating characteristic curve; Tregs, regulatory T cells; TCGA, The Cancer Genome Atlas; TACE, transcatheter arterial chemoembolization; TIDE, tumor immune dysfunction and exclusion; TME, tumor microenvironment; TMB, tumor mutation burden.
