# Supplementary figures and images for: Glutamine Metabolism Scoring Predicts Prognosis and Therapeutic Resistance in Hepatocellular Carcinoma
Source: Pathol Oncol Res. 2021 Dec 14;27:1610075. doi: 10.3389/pore.2021.1610075 (PMC8724684; doi:10.3389/pore.2021.1610075)

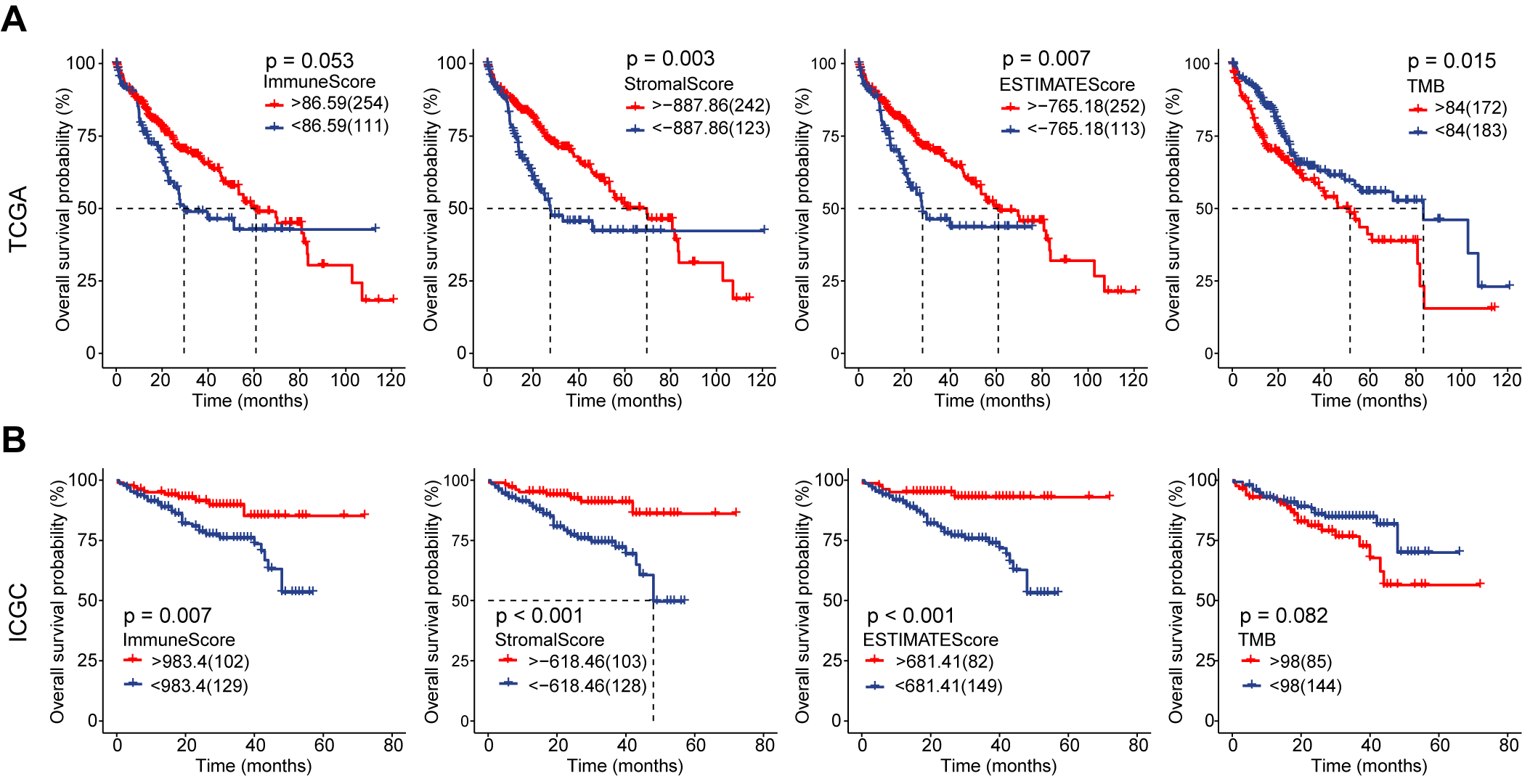

Supplement: Supplementary file 3 [file Image2.TIF]

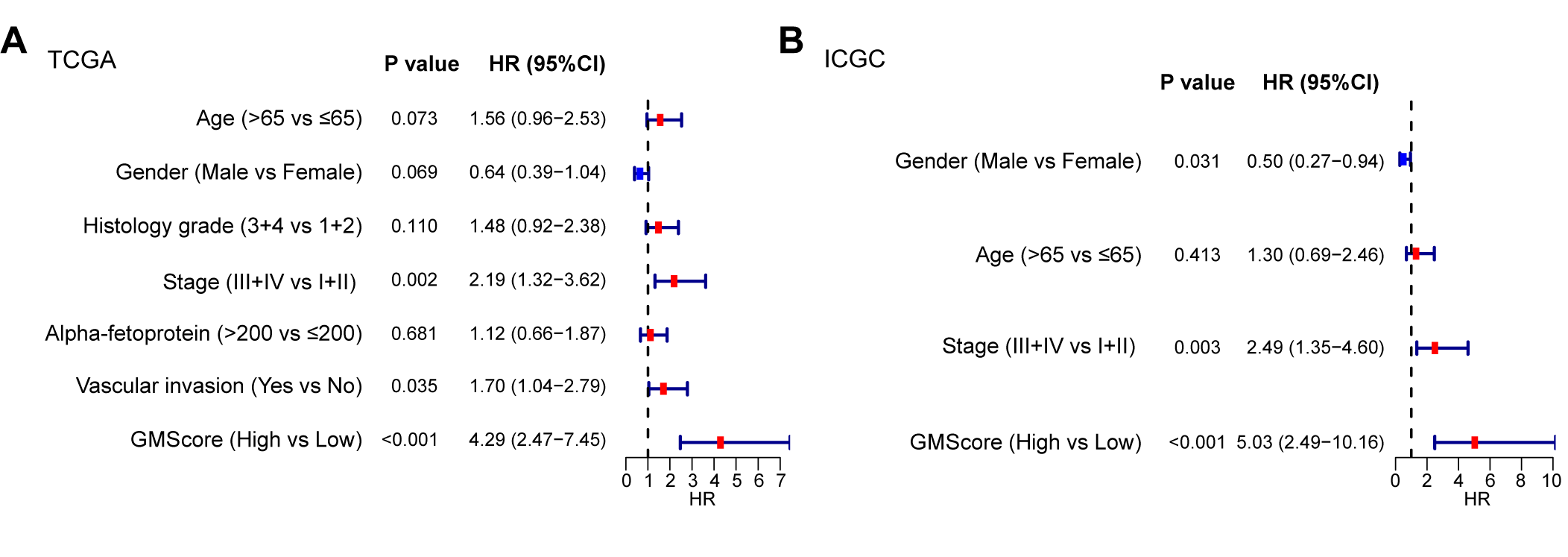

Supplement: Supplementary file 5 [file Image1.TIF]
