# Supplementary material for: Glutamine Metabolism Scoring Predicts Prognosis and Therapeutic Resistance in Hepatocellular Carcinoma
Source: Pathol Oncol Res. 2021 Dec 14;27:1610075. doi: 10.3389/pore.2021.1610075 (PMC8724684; doi:10.3389/pore.2021.1610075)
Supplement: Supplementary file 6 [file Table2.DOCX]

**Table S2.** Patient characteristics

| **Characteristic** | **TCGA (%)** | **ICGC (%)** | **P value** |
| --- | --- | --- | --- |
| Age(median,range) | 60 (16-90) | 67 (31-89) | <0.001^*^ |
| Gender | | | |
| Female | 119 (32.6%) | 61 (26.4%) | 0.108 |
| Male | 246 (67.4%) | 170 (73.6%) |  |
| Stage | | | |
| I+II | 254 (69.6%) | 141 (61.0%) | <0.001 |
| III+IV | 87 (23.8%) | 90 (39.0%) |  |
| Unknown | 24 (6.6%) | 0 (0.0%) |  |
| Histology grade | | | |
| G1+G2 | 230 (63.0%) | NA |  |
| G3+G4 | 130 (35.6%) | NA |  |
| Unknown | 5 (1.4%) | NA |  |
| Alpha-fetoprotein | | | |
| ≤200ng/ml | 201 (55.1%) | NA |  |
| >200ng/ml | 75 (20.5%) | NA |  |
| Unknown | 89 (24.4%) | NA |  |
| Vascular invasion | | | |
| No | 205 (56.2%) | NA |  |
| Yes | 106 (29%) | NA |  |
| Unknown | 54 (14.8%) | NA |  |

^*^Student’s *t*-test

TCGA: The Cancer Genome Atlas; ICGC: International Cancer Genome Consortium; NA: not available.
